# A Prospective Phase II Study of Automated Non-Coplanar VMAT for Recurrent Head and Neck Cancer: Initial Report of Feasibility, Safety, and Patient-Reported Outcomes

**DOI:** 10.3390/cancers14040939

**Published:** 2022-02-14

**Authors:** Kaley E. Woods, Ting Martin Ma, Kiri A. Cook, Eric D. Morris, Yu Gao, Ke Sheng, Amar U. Kishan, John V. Hegde, Carol Felix, Vincent Basehart, Kelsey Narahara, Zhouhuizi Shen, Stephen Tenn, Michael L. Steinberg, Robert K. Chin, Minsong Cao

**Affiliations:** 1 Department of Radiation Oncology, University of California, Los Angeles, Los Angeles, CA 90095, USA; kaley.woods@med.usc.edu (K.E.W.); tma@mednet.ucla.edu (T.M.M.); edmorris@mednet.ucla.edu (E.D.M.); yugao@mednet.ucla.edu (Y.G.); ksheng@mednet.ucla.edu (K.S.); aukishan@mednet.ucla.edu (A.U.K.); jhegde@mednet.ucla.edu (J.V.H.); cfelix@mednet.ucla.edu (C.F.); vbasehart@mednet.ucla.edu (V.B.); kcfong@mednet.ucla.edu (K.N.); zshen@mednet.ucla.edu (Z.S.); stenn@mednet.ucla.edu (S.T.); msteinberg@mednet.ucla.edu (M.L.S.); 2 Department of Radiation Oncology, University of Southern California, Los Angeles, CA 90033, USA; 3 Department of Radiation Oncology, Oregon Health & Science University, Portland, OR 97239, USA; cooki@ohsu.edu

**Keywords:** HyperArc, SBRT, reirradiation, recurrent head and neck cancer, non-coplanar VMAT

## Abstract

**Simple Summary:**

The delivery of higher radiation doses has been shown to increase local control, and ultimately survival, for head and neck cancer patients, but highly conformal dose distributions are necessary to minimize normal tissue toxicity. Varian’s HyperArc non-coplanar automated treatment planning and delivery technique has been shown to improve dose conformity for intracranial treatment, but its safety and efficacy for head and neck cancer treatment has yet to be verified. This study evaluates the initial results of a prospective clinical trial using HyperArc for recurrent head and neck cancer patients. We demonstrated that HyperArc can enable significant tumor dose escalation compared to conventional volumetric modulated arc therapy (VMAT) planning while minimizing the dose to organs at risk. Treatment delivery was feasible and safe, with minimal treatment-related toxicities and positive patient-reported quality of life measures.

**Abstract:**

This study reports the initial results for the first 15 patients on a prospective phase II clinical trial exploring the safety, feasibility, and efficacy of the HyperArc technique for recurrent head and neck cancer treatment. Eligible patients were simulated and planned with both conventional VMAT and HyperArc techniques and the plan with superior dosimetry was selected for treatment. Dosimetry, delivery feasibility and safety, treatment-related toxicity, and patient-reported quality of life (QOL) were all evaluated. HyperArc was chosen over conventional VMAT for all 15 patients and enabled statistically significant increases in dose conformity (R50% reduced by 1.2 ± 2.1, *p* < 0.05) and mean PTV and GTV doses (by 15.7 ± 4.9 Gy, *p* < 0.01 and 17.1 ± 6.0 Gy, *p* < 0.01, respectively). The average HyperArc delivery was 2.8 min longer than conventional VMAT (*p* < 0.01), and the mean intrafraction motion was ≤ 0.5 ± 0.4 mm and ≤0.3 ± 0.1°. With a median follow-up of 12 months, treatment-related toxicity was minimal (only one grade 3 acute toxicity above baseline) and patient-reported QOL metrics were favorable. HyperArc enabled superior dosimetry and significant target dose escalation compared to conventional VMAT planning, and treatment delivery was feasible, safe, and well-tolerated by patients.

## 1. Introduction

The management of patients with recurrent head and neck cancer (HNC) is a known challenge. Up to 50% of HNC patients experience locoregional recurrence within the first three years, which is most often the cause of death for this patient population [1,2]. Although surgery is generally the preferred option for these patients in the absence of distant metastatic disease [3], this is often infeasible due to comorbidities or anatomical considerations. Re-irradiation, often with concurrent systemic therapy, is the next best option for many of these patients, and as an adjuvant treatment for patients who do receive surgical resection [4]. However, conventionally fractionated treatment regimens have historically resulted in high toxicity rates and even treatment-related deaths, with 5-year survival rates of 15–35% [4,5,6,7,8].

There is a growing body of evidence to support the improvements in local control rates achieved with higher tumor doses [5,9,10]. The use of stereotactic body radiation therapy (SBRT) for re-irradiation enables the delivery of higher dose treatments with less dose spillage to previously irradiated tissues. Initial studies in recurrent HNC patients receiving re-irradiation with SBRT show improvement over conventional techniques, with one-year local control rates over 70% for small tumors, median overall survival times of up to 15 months, and acute grade ≥ 3 toxicity rates of around 20% [11,12,13,14,15].

However, even with SBRT, the long-term toxicity rates are high, and the outcomes are poor for patients with larger tumors (gross tumor volumes > 25 cm^3^) [13,14,16]. Since head and neck lesions are typically proximal to multiple critical structures, the safely deliverable prescription dose is usually limited by normal tissue toxicity. Further increasing dose conformality is, therefore, critical for achieving significant dose escalation in recurrent HNC treatment. It is well known that the incorporation of non-coplanar beam angles can significantly improve dose conformality and normal tissue sparing [17,18,19,20,21]. However, the manual selection of non-coplanar beam angles is unintuitive and increases the risk of collision between the patient and the treatment machine. Complicated couch and gantry motion resulting from non-coplanar beams often leads to prolonged treatment times as well [22,23]. To address these issues, the HyperArc technique (Varian Medical Systems, Palo Alto, CA, USA) was developed for cranial stereotactic radiosurgery treatments. HyperArc enables automated non-coplanar beam selection for superior dosimetry as well as automated gantry and couch motion for high-efficiency treatment delivery. It was demonstrated in a previous dosimetric comparison study that the HyperArc automated non-coplanar treatment technique can achieve significantly more conformal dose distributions and higher target doses than conventional coplanar volumetric modulated arc therapy (VMAT) planning for sites in the head and neck [24]. However, the safety, treatment time, and efficacy of this advanced treatment technique need to be further assessed in the clinical setting.

To evaluate the feasibility and safety of using the HyperArc technique for dose-escalated recurrent HNC treatment, as well as the effects on local control and toxicity, we developed a prospective phase II clinical trial (NCT03892720). Since the HyperArc technique and the corresponding immobilization devices were originally designed for intracranial treatment, the setup uncertainty and patient intrafraction motion for head and neck targets were evaluated, as well as the treatment planning, quality assurance, and delivery workflows. This study presents the dosimetric results, treatment delivery details, initial toxicity results, and patient-reported QOL outcomes for the first 15 patients on this trial.

## 2. Methods

### 2.1. Patient Characteristics

Between February 2020 and February 2021, 15 patients with histologically confirmed recurrent HNC who had received previous head and neck radiation therapy (RT) and had a maximum tumor (or tumor bed) diameter of 5 cm were enrolled in this prospective nonrandomized phase II trial. The trial was approved by the institutional review board (IRB# 18-001247). The primary objectives of this study are to evaluate the safety and feasibility of using HyperArc for head and neck targets, as well as the toxicity and local control of patients receiving dose-escalated SBRT re-irradiation with HyperArc for recurrent HNC treatment. Primary endpoints of the study include the local control rate of the treated tumor at one year and the percent of patients with acute or chronic grade ≥ 3 treatment-related toxicity at one year. Overall survival, progression-free survival, and locoregional progression-free survival are secondary endpoints. 

**Figure 1:**
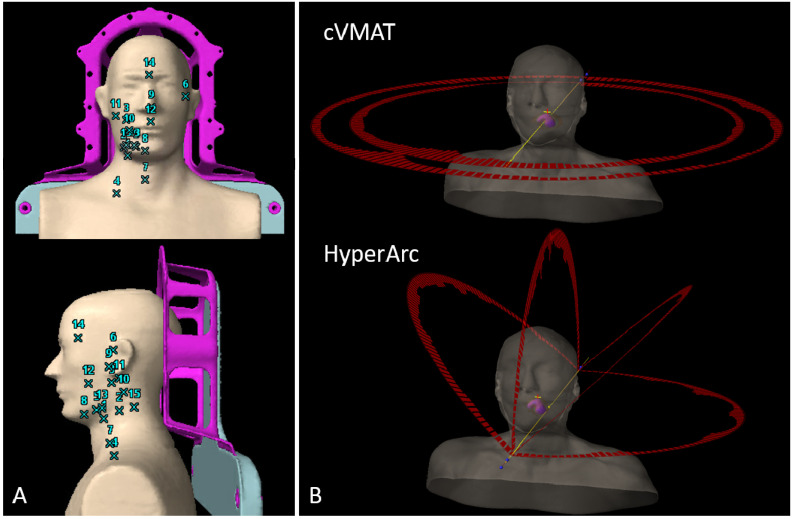
Target volume locations and beam arrangements for treatment planning. (**A**) PTV centroids illustrated for all 15 patients, localized with respect to the Qfix Encompass head frame used for all patients; (**B**) Beam arrangements for the cVMAT and HyperArc plans for one representative patient in the study (Patient 12).

In this work, we report the results for the first 15 patients of the study, treated between March 2020 and March 2021, whose characteristics are given in Table 1. The mean age was 68.7 years at the time of enrollment, with a mean planning target volume (PTV) lesion volume of 18.1 ± 18.2 cm^3^ (range 1.0–77.1 cm^3^). The patients received a prior dose of 65.6 ± 4.8 Gy (mean ± standard deviation), and two patients on the study received an additional prior course of radiation (see Table 1 caption). Twelve out of 15 patients had squamous cell carcinoma, with treatment sites ranging from the orbit to the supraclavicular nodes. The locations of the PTV centroids for all patients are illustrated in Figure 1A, localized with respect to the head immobilization frame. Five patients received concurrent pembrolizumab immunotherapy with SBRT, and none received concurrent chemotherapy.

### 2.2. Simulation and Treatment Planning

During the CT simulation, patients were immobilized using the Qfix Encompass SRS Immobilization System (Qfix, Avondale, PA, USA) and scanned on a Philips Brilliance Big Bore CT scanner (Philip Healthcare, Andover, MA, USA) with a spatial resolution of 1 × 1 × 1.5 mm^3^. PET/CT and MRI scans were also obtained if not performed in the six weeks prior to CT simulation. Based on these three scans, the gross tumor volume (GTV) or clinical target volume (CTV) was delineated for unresectable or resected patients, respectively. A planning target volume (PTV) was created by adding a 2 mm isotropic margin to the GTV or CTV to account for any treatment setup error. Dose was prescribed to cover 95% of the PTV unless the target coverage had to be sacrificed to meet clinical normal tissue constraints.

All planning was carried out with the Varian Eclipse treatment planning system (version 15.6) for a TrueBeam™ STx with a High Definition 120 MLC (6X-FFF, 1400 MU/min) (Varian Medical Systems, Palo Alto, CA, USA). Each patient was initially planned using conventional VMAT planning methods (RapidArc, Eclipse), hereafter referred to as cVMAT, to a standard prescription dose of 40 Gy in 5 fractions, with hotspots encouraged in the center of the target. Most cVMAT plans utilized two full coplanar arcs, with slightly non-coplanar, manually selected arcs added in a few cases for improved dosimetry. These plans aimed to minimize all organ-at-risk (OAR) doses, with strict maximum dose constraints (defined as the dose to 0.035 cm^3^) placed on the brainstem (<8 Gy), spinal cord (<8 Gy), mandible (<20 Gy), larynx (<20 Gy), and skin (<39.5 Gy). These conservative dose constraints were used to account for uncertainties in terms of prior dose distributions, image registration accuracy, and the degree of normal tissue repair following previous radiation treatment. 

Each case was then re-planned using Varian’s HyperArc technique, which automatically selects from five half arcs at couch angles of 0°, 45°, 270°, or 315° with optimized collimator rotation. The cVMAT and HyperArc beam arrangements for one example patient in the study are shown in Figure 1B. For each HyperArc plan, the goal was to achieve clinically comparable OAR doses to the cVMAT plan while escalating the prescription dose up to 55 Gy. Central hotspots were encouraged for the HyperArc plans as well, and both plans were created by the same experienced dosimetrist or physicist.

Plans were evaluated on the basis of OAR doses, target coverage, and conformity, and the superior plan was selected by the treating radiation oncologist. These dosimetric parameters were all compared between the two plan types, including the R50% (50% prescription isodose volume divided by the PTV volume) and gradient measure (difference between the equivalent sphere radii of the 50% and 100% isodose volumes) as metrics to assess the plan conformity.

Prior to treatment, each HyperArc plan was measured with the PTW Octavius phantom (PTW, Freiburg, Germany) for quality assurance, and a gamma criterion of 3%/2 mm dose deviation was used for this comparison with a 95% passing rate threshold [25]. A modified verification plan was delivered with all couch angles set to zero since couch rotations introduce the risk of damaging radiation-sensitive electronics within the Octavius phantom.

### 2.3. Treatment Delivery

Before the first fraction of each treatment course, a dry run was performed by the therapists in the treatment room to check for potential collisions before proceeding with the automated treatment delivery. A cone-beam CT scan (CBCT) was acquired to enable precise patient setup and reviewed by a radiation oncologist prior to beam delivery for each fraction. To assess intrafraction motion for HyperArc head and neck treatment, a post-treatment CBCT was also acquired for each fraction. A radiation oncologist performed a rigid registration on these two images, focusing on the PTVs, to determine the intrafraction patient shifts. 

These measured shifts were then used to calculate the necessary PTV margins for adequate target coverage. The systematic error (∑) in each direction (translation and rotation) was calculated as the standard deviation of the mean shifts for each patient for all five treatment fractions, and the random error (σ) was calculated as the root mean square of the standard deviations across all patients. The PTV margins were then calculated using the formula from van Herk et al. [26], developed to ensure a minimum CTV dose of ≥95% of the prescription dose for 90% of patients.

The treatment delivery times were also compared between the two plan types using the beam delivery times recorded in the record and verify system for the treated plans and manual measurements in quality assurance delivery mode for the untreated plans. The delivery time was defined from the start of the first beam to the end of the last, excluding patient setup and imaging. The image guidance time, defined as the time from the start of the CBCT to the start of the first beam delivery, was also collected for each HyperArc fraction. This includes the time for the physician and physicist to come to the machine and review the patient images and setup. 

### 2.4. Clinical Assessment

Patients received a clinical examination one month after treatment completion, PET/CT and MRI scans three months post-treatment, and MRI scans every subsequent three months until one year, at which point patient participation in the trial was complete. Clinician-reported measurements (CROMs) and patient-reported outcome measurements (PROM) were assessed at baseline and at 1, 3, 6, 9, and 12 months after the start of RT to evaluate treatment-related toxicity and patient quality of life (QOL).

#### 2.4.1. Toxicity Measurement

CROMs were scored using Common Terminology Criteria for Adverse Events (CTCAE) v.4.0. We systematically scored the incidence and grading of symptoms including, but not limited to, dysphagia, dry mouth, dysgeusia, mucositis, pain, ulcers, and dermatitis. Early toxicity is defined as toxicity occurring within or at 3 months of the start of treatment and late toxicity is defined as toxicity occurring any time after 3 months of the start of treatment. A Pocock-type sequential stopping boundary for toxicity was used to determine if at any point the patient accrual needed to be halted early due to excessively high toxicity [27]. 

#### 2.4.2. Quality of Life Measurement

QOL was measured using the Functional Assessment of Cancer Therapy Head and Neck (FACT-H&N) questionnaire version 4, as well as the University of Washington Quality of Life Questionnaire (UW-QOL) version 4. FACT-H&N version 4 is a 39-item previously validated questionnaire consisting of a 27-item oncology-specific QOL instrument (FACT-G) followed by a 12-item HNC-specific subscale (HN) [28,29,30]. FACT-G includes 27 questions in four domains—physical well-being (7), social/family well-being (7), emotional well-being (6), and functional well-being (7). Patients rated each question from 0 to 4 on a Likert scale, with 0 as “not at all” and 4 as “very much.” Scores were calculated separately for each domain and an unweighted aggregate score was calculated to yield the FACT-H&N Total score (FACT-G+ HN). A higher score indicates better QOL with a maximum score of 148 (37 total scorable questions, 4 points each) reflecting the best possible QOL. The maximal score for each domain is as follows: Physical—28, Social—28, Emotional—24, Functional—28, HN—40. A clinically significant change in FACT-H&N score is represented by an increase of about 6.2 or a decrease of about 12.4 [31].

The UW-QOL version 4 is a 16-item (17 if including a free-text question at the end) patient-reported scale measuring health-related QOL specifically for HNC patients [32,33,34,35]. It consists of 12 domain-specific questions, an importance rating among 12 domains, and three questions on global QOL. The first 12 questions evaluate the following domains: pain, appearance, activity, recreation, swallowing, chewing, speech, shoulder, taste, saliva, mood, and anxiety. Each question is based on discrete ordinal responses whose scores range from 0 (dysfunctional or lowest level) to 100 (normal or highest level). A composite UW-QOL score was calculated as the mean of the 12 individual domain scores, with a maximum possible score of 100. The UW-QOL has been extensively validated [36,37,38]. 

### 2.5. Statistical Analysis

A paired, two-tailed *t*-test was used to determine if the differences in delivery time, conformity, or target and OAR dose were statistically significant between cVMAT and HyperArc. The one-way analysis of variance (ANOVA) was used to determine whether there were any statistically significant differences between the means of QOL scores at different time points. Statistical significance was set at *p* < 0.05. 

## 3. Results

The cohort had a median follow-up time of 12 months (interquartile range 6–13 months), with eight patients completing the 12-month study follow-up period. The median interval from initial RT course to the current re-irradiation course was 33 months (interquartile range 17–62 months, range 3–179 months).

### 3.1. Dosimetry

**Figure 2:**
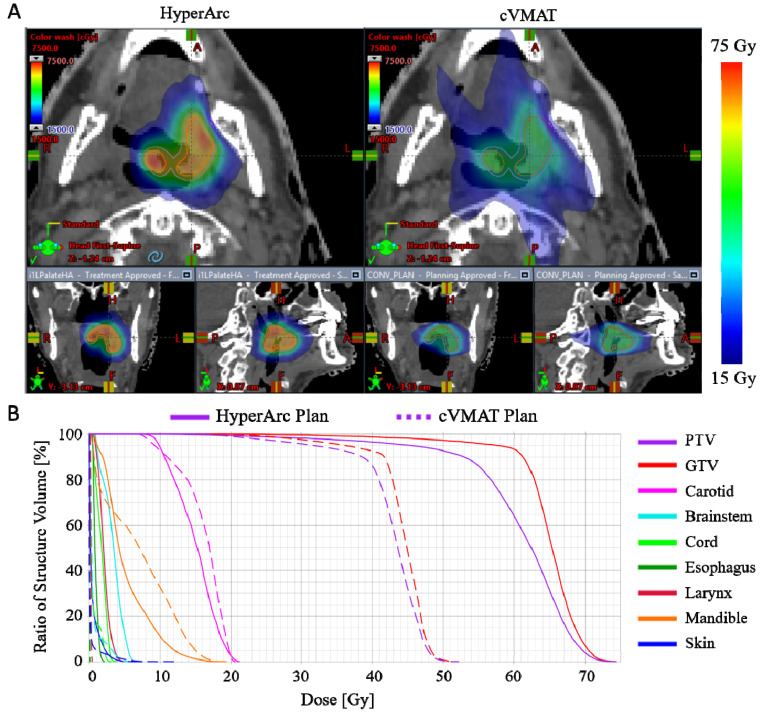
Dose comparison between HyperArc and cVMAT plans for one representative patient in the study (Patient 12). (**A**) Isodose distributions in color wash (GTV shown in orange and PTV shown in blue); (**B**) Dose volume histograms for the HyperArc (solid line) and cVMAT (dashed line) plans.

For all 15 patients, the HyperArc plan was chosen over the cVMAT plan for treatment because of superior dose conformity and target coverage (Table 2 and Figure 2). The HyperArc plans were significantly more conformal than the cVMAT plans, with a statistically significant difference in gradient measure (0.7 ± 0.1 vs. 0.8 ± 0.2, *p* < 0.001) and R50% (2.9 ± 1.3 vs. 4.1 ± 3.0, *p* = 0.039). The HyperArc plans achieved an average escalation in mean GTV and PTV doses of 17.1 Gy (58.2 vs. 41.1, *p* < 0.001) and 15.7 Gy (56.3 vs. 40.6, *p* < 0.001), respectively. The maximum OAR doses were all below the planning constraints, as shown in Table 2. Although there was a statistically significant increase in maximum dose for some OARs with the HyperArc plans, these were mostly for low-dose OARs receiving < 5 Gy total, with all average maximum dose differences below 2.5 Gy. 

### 3.2. Treatment Delivery

The treatment delivery times, averaged over all five fractions for the HyperArc plans, are given in Table 2. Although the difference in delivery times between the two plan types was statistically significant due to the larger number of beams and multiple couch rotations with HyperArc, the average increase in delivery times was only 2.8 min. The average image guidance time for all patients was 8.7 min for the first fraction and 4.9–5.9 min for subsequent fractions. The first patient in the study had the longest image guidance times of ~27 min for two of the fractions, and when this patient is excluded, the average time for all fractions decreases from 6.1 to 5.5 min.

The intrafraction motion measurements and PTV margins calculated with the van Herk formula are given in the Appendix A, Table A1. The mean translational shifts were all ≤ 0.5 ± 0.4 mm, and rotational shifts were all ≤ 0.3 ± 0.1°. The calculated systematic and random error was ~0.4 mm (0.1°) and ~0.7 mm (0.3°), respectively, yielding PTV margins of ≤ 1.5 mm for all three directions. There were only four fractions (5.3%) with shifts greater than 2 mm out of all 75 fractions analyzed. Two of these fractions (with shifts of 5.3 and 2.3 mm laterally and 3.4 mm longitudinally) were for the first patient treated on the trial, who showed a change in neck positioning during treatment due to suboptimal fitting of the immobilization mask. The other two (with shifts of 2.2 mm laterally and 5.0 mm vertically) occurred with the eighth patient, treated for a tongue lesion.

### 3.3. Toxicity and Patient-Reported Outcomes

**Figure 3:**
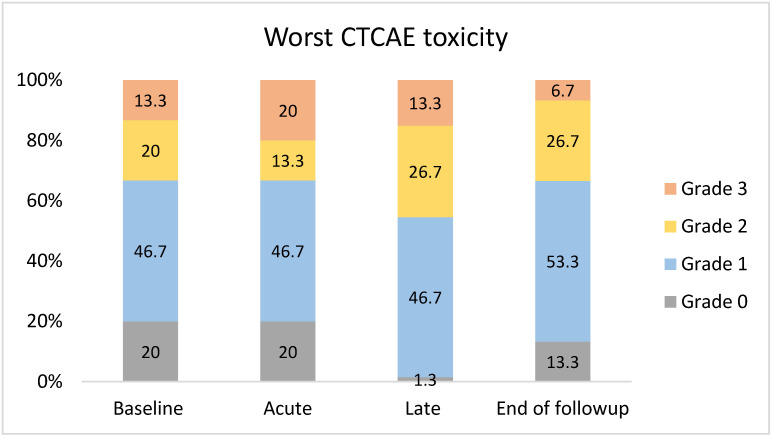
Clinician-reported early (≤3 months after start of treatment) and late (>3 months after start of treatment) toxicity, classified according to Common Terminology Criteria for Adverse Events criteria.

Acute and late grade 2 toxicities were 13.3% and 26.7%, and grade 3 toxicities were 20.0% and 13.3%, respectively, as shown in Figure 3. At the end of follow-up, grade 2 and 3 toxicities were 26.7% and 6.7%, respectively. Grade 2 and 3 pre-treatment head and neck morbidities were 20.0% and 13.3%, respectively. Acute and late grade 2 toxicities exceeding baseline were both 6.7% and remained at 6.7% at the end of the follow-up. There was no grade 3 toxicity exceeding baseline at the end of follow-up. Three patients experienced grade 3 acute toxicity (patients #2, #13 and #15, Table 1). However, in two of them (patients #2 and #15), the grade 3 toxicity was present at baseline (dysphagia and dysphagia and dry mouth, respectively). Patient #13 developed grade 3 oral mucositis three weeks after the completion of SBRT, which resolved within one month. 

**Figure 4:**
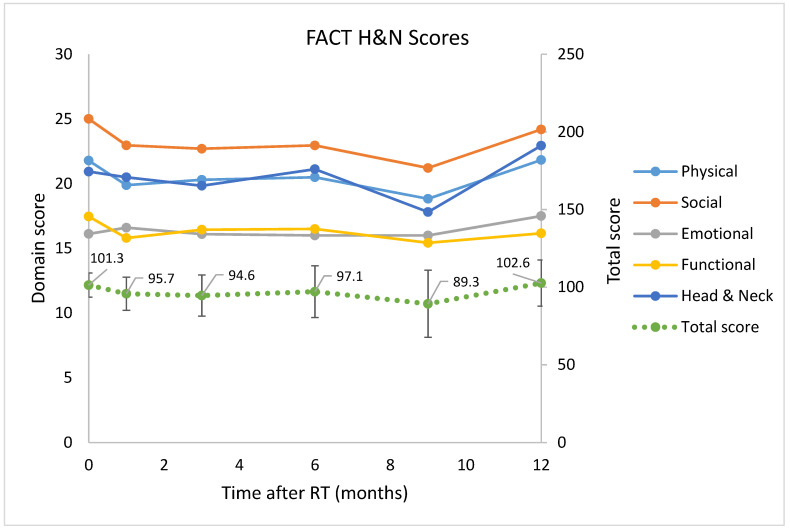
FACT H&N subdomain and total scores at baseline and at each follow-up time post-treatment.

There was an initial decline in mean FACT H&N subdomain scores and total scores post-RT, but these scores recovered to pre-treatment levels after 12 months (mean total scores were 101.3 at screening and 102.6 at 12 months after treatment), as shown in Figure 4. There was no statistically or clinically significant change in the mean HNC-specific subscale score or the FACT H&N total score at any time point after RT (*p* = 0.91 and 0.80, respectively). 

**Figure A1:**
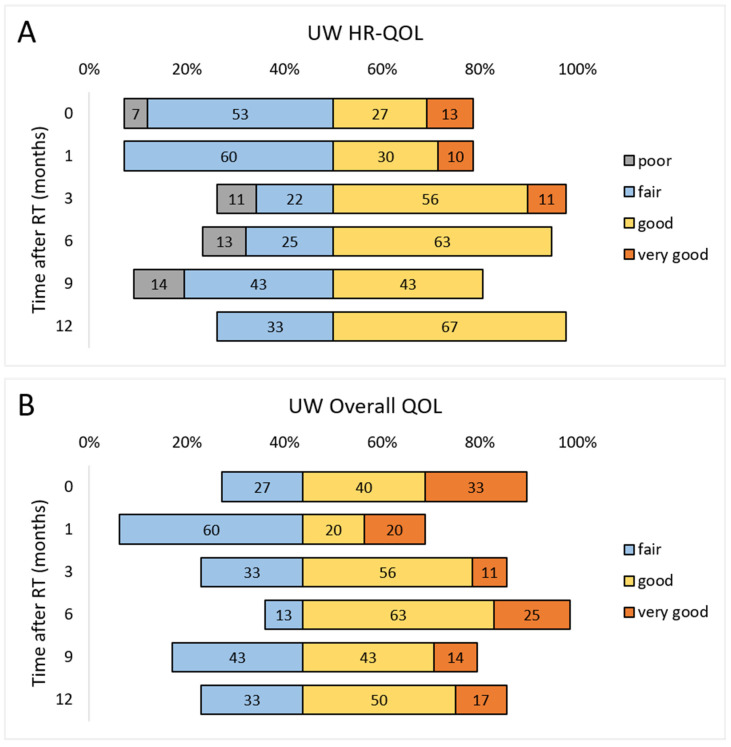
Patient-reported quality of life scores based on the UW-QOL questionnaire at baseline and at 1, 3, 6, 9 and 12 months after treatment. (**A**) Health-related QOL scores; (**B**) Overall QOL scores.

The favorable toxicity profile was confirmed by the UW-QOL questionnaire, the results of which are shown in Appendix A, Figure A1. At 12 months post-RT, 67% of patients reported that both their health-related QOL and overall QOL were good or very good, and no patients reported poor QOL. There was no statistically or clinically significant change in the mean composite score of UW-QOL (first 12 domains) (*p* = 0.95), HR-QOL score (*p* = 0.94), or overall QOL score (*p* = 0.61) at any time point post-RT. 

## 4. Discussion

The present study demonstrated that compared to conventional VMAT plans, the HyperArc plans achieved more conformal dose distributions and significantly higher target doses with favorable physician-scored toxicity profiles and patient-reported QOL outcomes. To our knowledge, this is the first prospective phase II study on dose-escalated SBRT utilizing non-coplanar VMAT in the re-irradiation setting for HNC. Previous dosimetric studies have shown that significant dose reduction to surrounding OARs and improved target dose coverage can be achieved with non-coplanar IMRT/VMAT [17,23,39,40]. However, one key difference is that the current study significantly escalated the dose to the targets beyond the prescription dose of 40 Gy to a mean PTV dose of 56.3 Gy while largely maintaining the dose to the OARs, as dose escalation has been correlated with improved locoregional control during irradiation of head and neck tumors [13,41]. In our previous HyperArc dosimetry study, increasing the GTV dose by 10.8 ± 4.4 Gy with HyperArc increased tumor control probability (TCP) from 43.8 ± 21.1% with conventional VMAT to 61.4 ± 42.5% [24]. In the current study, we increased the GTV dose by 17.1 ± 6.0 Gy, which correlates to a greater TCP. Admittedly, some structures superior or inferior to the PTV, such as the optic nerves and cochleae, received a slightly higher maximum dose with HyperArc due to the non-coplanar beam arrangements utilized. Because the absolute maximum doses to these structures were all <5 Gy (1 Gy/fraction), the greater maximal doses are clinically insignificant. For higher dose (i.e., more proximal) structures such as the mandible and larynx, the average maximum dose differences were around 2 Gy, i.e., less than 50 cGy per fraction. This is consistent with the report by Gayen et al., who showed that the low dose spillage volume of V5Gy was marginally increased with non-coplanar VMAT, although V10Gy was reduced [40].

No notable setup or delivery issues were observed with HyperArc treatment. Although the first patient in the study had relatively long image guidance times for two fractions, this time decreased significantly for subsequent patients. This was likely the result of a learning curve for the therapists and oncologists since this was the first patient to ever be treated with the HyperArc technique at this clinic.

Although treatment delivery times were slightly longer for HyperArc than conventional VMAT treatments due to the greater number of fields and couch rotations, this two-minute average difference is not clinically significant. The HyperArc treatments easily fit into the normal machine schedule with 15- to 20-min time slots. The HyperArc planning module also provides a virtual collision dry run function that can replace the actual in-room test, further reducing the treatment time. The HyperArc treatment planning process also has several automated features that likely enable shorter planning times compared to conventional VMAT, although this would require further investigation. The robust optimization algorithm used in HyperArc planning was shown to reduce inter-operator variability for cranial SRS planning [42]. A similar trend has been observed in this study, but the effectiveness of reducing planning iterations and variations warrants a future study. 

Since the existing HyperArc-compatible immobilization device is designed for intracranial targets and does not extend past the shoulders, one initial concern was whether this mask could adequately immobilize HNC patients with more inferior lesions. Nevertheless, the observed intrafraction motion was minimal, with mean shifts of about 0.5 mm and a calculated necessary PTV margin of about 1.5 mm, less than the 2 mm margin used in this study. Three of the five larger shifts (>2 mm) occurred with the first patient on the trial. This can most likely be attributed to the therapists’ learning curve with the HyperArc mask; for subsequent patients, they expressed greater confidence and were able to fit the mask more securely. This was also reflected by the minimal intrafraction motion seen for all other patients except one, who was being treated for a tongue lesion. In this case, the patient had lateral and/or vertical motion of over 2 mm for two fractions due to insufficient tongue compression, which will be addressed for future patients. 

The HyperArc treatment was well-tolerated by patients, with minimal toxicity. Of the three grade 3 toxicities reported, two were present at the time of study enrollment, and only one developed de novo after the SBRT but resolved quickly. This compared favorably to the grade 3 toxicity of 0–31% reported in the literature with SBRT re-irradiation [41,43,44], although in those studies, many received concurrent cetuximab with radiation, which is not the case in this study. The patient-reported QOL was also favorable, with no significant difference between baseline and 12 months post-treatment and with only a slight initial decrease at one-month follow-up.

Another potential treatment alternative for head and neck re-irradiation is proton therapy, which has also been shown to significantly reduce normal tissue doses compared to conventional photon therapy [45,46,47]. In a multicentric review by Eekers et al., when normalized to the same target dose, intensity-modulated proton therapy enabled reductions of ~20–65% to the larynx, mandible, oral cavity, brainstem, and cord compared to conventional VMAT [45]. Similarly, in this study, we demonstrated an average dose escalation of approximately 40% with HyperArc while maintaining comparable doses to these organs-at-risk. This increased normal tissue sparing has the potential to reduce treatment-related complications. In a retrospective review of patients receiving oropharyngeal re-irradiation at MD Anderson Cancer Center, the patients receiving proton therapy experienced lower grade ≥3 toxicity rates (27%) compared to SBRT (32%) or IMRT (41%) [48]. Other proton head and neck re-irradiation studies have published acute grade ≥3 toxicity rates of 3–30%, and late toxicity rates of 16–25% [49,50,51,52]. Therefore, the toxicity rates observed from HyperArc treatment thus far compare very favorably to those published for re-irradiation with proton therapy. Despite its inherent dosimetric advantages, the widespread adoption of proton therapy is limited by cost and accessibility. HyperArc, by contrast, is a widely available treatment technique using existing C-arm linear accelerators.

Although these initial results are promising, this study is limited by the small patient cohort. Study enrollment is still in progress, with a target sample size of at least 45 patients. The reported follow-up times are also short, with only eight patients reaching the one-year follow-up assessment. Tumor control data are therefore limited, but early results are encouraging. After further patient enrollment and follow-up are completed, more extensive clinical outcome results will be reported.

## 5. Conclusions

In this work, we presented the initial results for the first 15 subjects on a phase II study investigating the use of the HyperArc automated non-coplanar treatment planning and delivery technique for recurrent HNC patients. HyperArc enabled significant tumor dose escalation compared to conventional VMAT with comparable OAR sparing, and there were no concerns with the safety or efficiency of treatment delivery. The HyperArc treatment was also well-tolerated by patients, with low toxicity rates and favorable patient-reported QOL outcomes. Results for a larger cohort, as well as oncological outcome data, will be reported after further study progress.

## Figures and Tables

**Table 1:** Patient characteristics.

Pt	Age (years)	Sex	Initial Primary Tumor/RT Site	Prior Dose (Gy)	Interval between RT (months)	Recurrent Site	Histology	PTV Volume (cm^3^)	Concurrent Systemic Therapy
1	57	M	Left floor of mouth	66 ^1^	4	Right neck	SCC	77.1	Pembrolizumab
2	59	M	Right nasopharynx	70	19	Right neck	Undifferentiated carcinoma	4.9 * [2.7, 1.2, 1.0]	None
3	66	F	Left floor of mouth	66	3	Right neck	SCC	38.8 * [19.1, 19.6]	Pembrolizumab
4	70	F	Right oral tongue	60	5	Right supraclavicular	SCC	13.8	Pembrolizumab
5	80	M	Right base of tongue	72	76	Right base of tongue	SCC	9.5	None
6	60	M	Left parotid	66	15	Temporalis	Salivary ductal carcinoma	44.5	Trastuzumab emtansine
7	75	M	Floor of mouth	70	38	Inferior hypopharynx	SCC	2.5	None
8	71	M	Left oral tongue	54	127	Tongue	SCC	9.5	None
9	51	M	Right oral tongue	66	40	Palate, sphenoid	SCC	30.0 * [11.6, 18.4]	None
10	87	M	Right floor of mouth	66 ^2^	33	Left retropharyngeal LN	SCC	7.2	Pembrolizumab
11	73	M	Right base of tongue	66	29	Right parotid	SCC	24.6	None
12	77	M	Right base of tongue	70	179	Left soft palate	SCC	15.2	None
13	53	M	Left lateral tongue	Unk	168	Right oral cavity	SCC	13.5	Pembrolizumab
14	67	M	Right nasal passage	60	33	Left ethmoid Sinus	Adenocarcinoma	43	None
15	85	F	Hard palate	66	48	Right mandible	SCC	9.9	None

^1^ Patient received two prior courses of RT: left modified radical neck dissection with adjuvant radiation completed 6/2017 and bilateral neck dissection with re-irradiation completed 10/2019; SBRT with HyperArc completed 3/2020. ^2^ Patient received two prior courses of RT: composite mandibulectomy with adjuvant RT completed 1/2018 and SBRT re-irradiation completed 7/2020; SBRT with HyperArc completed 11/2020. * PTV volume consists of multiple lesions; given as total PTV volume, with individual lesion volumes in brackets. SCC: squamous cell carcinoma; Unk: prior radiation therapy prescription unknown; RT: radiation therapy.

**Table 2:** Delivery time and dosimetric statistics.

Delivery Time, Conformity, and Target Dose					
		cVMAT	HyperArc	Absolute difference	*p* value
Mean delivery time (min)		2.5 ± 0.7	5.2 ± 2.1	2.8 ± 2.2	<0.001 *
R50%		4.1 ± 3.0	2.9 ± 1.3	−1.2 ± 2.1	0.039 *
Gradient measure		0.8 ± 0.2	0.7 ± 0.1	−0.1 ± 0.1	<0.001 *
PTV mean (Gy)		40.6 ± 5.4	56.3 ± 9.1	15.7 ± 4.9	<0.001 *
PTV max (Gy)		48.0 ± 7.2	68.4 ± 8.8	20.4 ± 7.0	<0.001 *
GTV mean (Gy)		41.1 ± 6.5	58.2 ± 11.0	17.1 ± 6.0	<0.001 *
GTV max (Gy)		47.8 ± 7.4	68.4 ± 8.9	20.6 ± 7.1	<0.001 *
Maximum OAR Doses ^†^ (Gy)					
	Planning constraint	cVMAT	HyperArc	Absolute difference	*p* value
Larynx	≤20	3.6 ± 6.6	6.0 ± 6.1	2.4 ± 1.9	<0.001 *
Cord	≤8	3.7 ± 2.3	4.1 ± 1.7	0.4 ± 2.1	0.506
Mandible	≤20	11.4 ± 7.9	13.0 ± 6.3	1.6 ± 2.9	0.051
Brainstem	≤8	3.4 ± 4.6	4.7 ± 4.4	1.3 ± 2.4	0.047 *
Skin	≤39.5	23.0 ± 11.8	21.3 ± 12.8	−1.8 ± 2.2	0.009 *
Chiasm	≤8	1.6 ± 3.2	2.8 ± 3.3	1.2 ± 3.1	0.167
Right optic nerve	≤8	1.1 ± 2.5	3.5 ± 3.0	2.4 ± 2.8	0.005 *
Left optic nerve	≤8	2.9 ± 6.7	3.4 ± 4.4	0.5 ± 3.4	0.567
Right cochlea	≤25	0.8 ± 1.3	3.2 ± 2.5	2.3 ± 2.5	0.003 *
Left cochlea	≤25	1.7 ± 3.9	3.4 ± 4.5	1.7 ± 2.0	0.006 *

Delivery time and dose metrics are expressed as mean ± standard deviation. * Statistically significant difference (paired, two-tailed *t*-test, *p* < 0.05). ^†^ Calculated as the maximum dose to 0.035 cm^3^ of tissue.

## Data Availability

The data presented in this study are available on request from the corresponding author. The data are not publicly available in compliance with institutional policy.

## Appendix A

**Table A1:** Intrafraction motion measurements, error, and van Herk PTV margin calculations. Mean shifts (and standard deviation) calculated for all 5 fractions, averaged over all 15 patients.

	Translations (mm)			Rotations (°)		
	Lateral	Vertical	Longitudinal	Pitch	Yaw	Roll
Shift measurements	0.5 ± 0.4	0.4 ± 0.4	0.5 ± 0.3	0.2 ± 0.1	0.3 ± 0.1	0.2 ± 0.1
Systematic error	0.4	0.4	0.3	0.1	0.1	0.1
Random error	0.7	0.7	0.6	0.3	0.3	0.2
van Herk PTV margin	1.4	1.5	1.2	0.5	0.6	0.4
